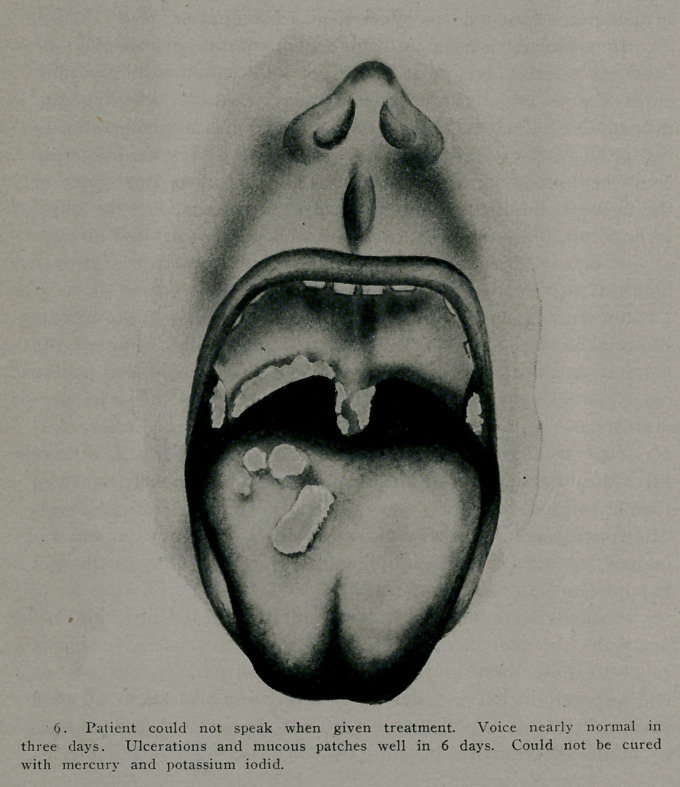# Salvarsan, or “606,” Ehrlich’s New Remedy for Syphilis

**Published:** 1911-03

**Authors:** Edgar M. Ballenger

**Affiliations:** Atlanta, Ga.; Lecturer on Genito-Urinery Diseases and Syphilis, Atlanta School of Medicine


### SALVARSAN, OR "606," EHRLICH'S NEW REMEDY FOR SYPHILIS.

By Edgar G. Ballenger, M. D., Atlanta, Ga.

*Lecturer on Genito-Urinary Diseases and Syphilis, Atlanta School of Medicine.*

It gives me pleasure to present before your Association an account of my visit to the German Clinics where salvarsan has been used most extensively and also to give my personal experiences with this wonderful remedy. Never in the history of medicine has such a potent therapeutic measure been discovered for the treatment of any disease, nor ever before has any scientific observation been so thoroughly tested before being submitted to general use. One of the most remarkable features is the nearly uniform reports as to the almost immediate disappearance of the spirochetes and the prompt healing action of salvarsan. It seems to matter not what the stage of the disease nor the extent (if the patients physical condition permits the treatment) splendid

1. Pain relieved in 24 hours. Ulcers cleared off in 48 hours, and healed in 5 days. Mucous patch under tongue, size of a quarter, healed in 4 days.

results are to be expected in the majority of patients. Parasyphilitic affections, not being due to active syphilitic infection, and chronic interstitial keratitis are an exception to this statement. The more active lesions, especially the late lesions, and the more malignant the disease, the better seem the results, or at least, the more apparent and wonderful. A few cases have been reported where the spirochetes were not affected by the remedy and no improvement followed its administration. Whether this is due to the germ being immune to arsenic or whether the patient is unable to develop properly anti-toxins has not been determined. The chemistry underlying Ehrlich's work is exceedingly interesting, but in such a short paper this and many other important phases of the work will have to be omitted. His study has been to find chemicals which had special affinity for the spirochetes and then to combine arsenic with these chemicals so that it could thus be forced on the organisms in sufficient quantity to kill them and yet do practically no harm to the body. Thousands of experiments had been made with most encouraging results and 605 different chemical combinations had been tried before "606," dioxydiamidoarsenobenzol dihydrochloride, or "salvarsan," was discovered. This last name is the trade name under which this remedy is patented and placed upon the market. Before the complicated method of its manufacture was known, we looked with considerable doubt upon the propriety of protecting a remedy of so great value to mankind with a patent, but as its manufacture is attended with many difficulties and must be done in an atmosphere of nitrogen or some inert gas to prevent the formation of a dangerous oxide of arsenic, and as each lot must be subjected to experimental tests to determine its nontoxicity as well as its potency, it seems much better to have it patented and manufactured by those who know how to test it and who feel responsible for its curative qualities and its success.

About 35,000 patients have now been treated with this remedy. Regarding the prompt healing of lesions and clearing up of all syphilitic manifestations in a remarkably short time, "606" may be said to be beyond the experimental stage. It is yet too early, however, to make predictions as to the ultimate curative results. Syphilis is known to be such a treacherous disease that

3. Thickly indurated chancre. Healed in 15 days.

at least 10 to 15 years or longer should elapse before the final word can be spoken.

The prompt manner in which is kills the spirochetes in infectious lesions will certainly immediately cause a reduction in the number of individuals infected unless the restraining influence of the fear of the disease, thus being lessened, causes an increase in licentiousness. That locomotor ataxia and syphilitic insanity will be greatly reduced seems already proven by the arrest of these affections in their *incipiency* by an injection of salvarsan.

Brain or cord diseases caused by a gummatous growth or by active syphilitic lesions of the mininges may be cured by this remedy, but if the destruction of the brain cells or the nerves has already occurred they cannot be replaced by any remedy. There was great fear at first that optic neuritis would be caused by "606" just as it followed in certain cases with atoxyl, but extensive use of salvarsan has shown that if wood alcohol be not used to bring it into solution there is no danger whatever of this

2. Greatly improved at end of week. Well in three weeks.

4. Ulcer on leg well 12 days after prolonged regular antisyphilitic treatment had failed.

complication. It is advised, however, that no patient be submitted to the treatment who has optic neuritis, retinitis or choroiditis unless these be of leutic origin and the increased danger under such circumstances explained to the patient. The remedy is also contra-indicated in serious organic affections of the heart, blood vessels, kidneys, liver, lungs, brain and spinal cord, and in gastric or bladder ulcer and in patients already moribund, although the reports show that patients have recovered when all hope of such a result was passed.

The Wassermann reaction in the majority of patients becomes negative in from 2 to 6 weeks and it is regarded necessary for this blood test to be made in every instance in order that an intelligent opinion may be expressed as to the complete success of the

5. Cured in 5 days.

treatment or whether a second or third injection is necessary. This test should be repeated in 6 and 12 months, even if there be no evidences of disease.

All the lesions of the primary, secondary and tertiary stages as a rule become clean and healthy promptly after the administration of salvarsan. If the ulceration or infiltration is extensive restitution to normal is slower, depending upon the extent of the disease. The lymphatic glands gradually become normal, the patients nearly always gain in weight and have a feeling of well-being which is quite surprising.

Ehrlich now recommends the intravenous injection of salvarsan in preference to the subcutaneous and intramuscular injections. He thinks the reports so far received indicate that this method is more likely to give permanent results and is preferable to other methods. While the technical difficulties of administering the intravenous injections are greater, the result is more prompt and there is less pain experiencd by the patient—this is especially true when, as sometimes happens, a local necrosis develops at the site of the subcutaneous injection. This is due either to infection or to the intense local reaction of the remedy. The pain following the subcutaneous or intramuscular injections at first was quite severe, but the modification of Wechselmann and the introduction of the neutral emulsion has greatly reduced the pain which now often does not require morphia or other anodynes. There are so many variation in the technique and as directions are to be found in every package of "606," we will not consider further this matter except to urge much preliminary study and the greatest of care in making these injections. The technique of preparing and administering the remedy should be just as perfect as if performing a laparotomy. What is of equal importance is the examination of the patient, first, to be sure the affection is syphilitic; second, to be sure there is no serious organic trouble that might render dangerous the possible by-effects of salvarsan.

A number of women nearing the end of pregnancy have received the treatment without interference with the normal delivery at full term.

Albumin and red blood cells may appear in the urine from a few days to a few weeks after the injection, but seem to me of no consequence except to act as a warning to administer "606" with great care or not at all to patients with serious kidney trouble, unless of luetic origin.

After the injection of "606," the parasites killed early evoke an active formation of antibodies, which assist the action of the drug. This is clearly shown by the fact that the milk of a syphilitic mother or wet-nurse at times after an injection contains enough of antitoxine to cure, or apparently cure the infant in the course of a week or so, and yet the amount of arsenic found in it is much too small to effect such a prompt cure.

It is thought advisable to give such infants an injections of "606" at a later date, as it is not yet known how well the milk cures them. Much care must be exercised in selecting the proper dose in infants, as enough of toxins has been liberated to cause the death of the infant, though no signs of arsenic poisoning could be found, nor would the same dose produce this effect in the milk of the mother or nurse, who has been treated, be taken at first to kill the majority of germs and nearly cure the disease.

Beneficial effects are usually noticeable after from 24 to 48 hours, if the treatment is to be successful.

In passing many times through the wards at the Virchow Hospital, I was impressed with the apparent well-being of the patients and with the scarcity of active syphilitic lesions—these being present only in the recently admitted patients. The temperature charts were as a rule about normal, sometimes there was a rise of from one to three degrees Fahrenheit for a few days after the injection, rarely the temperature went higher, but even then it remained so for a very short time only. The pulse was often quickened for a few days but in the average patient remained below 85. Very high blood pressure is at present thought to contraindicate the use of "606."

Patients were seen with masses at the site of injection, beneath the shoulder blade, varying in size from those of insignificance to those three inches in diameter and perhaps one or one and one-half inches in thickness. Sloughing had occurred in a small percentage of patients, but did not appear to hinder the cure of luetic affections.

The salvarsan should be prepared and injected immediately after the glass ampul, in which it is contained, has been opened. Rectal and bladder irritation has been found to follow occasionally when too much time has been taken in preparing it, thus permitting oxidation.

Marschalko has recently warned that the subcutaneous and intramuscular injections of "606" have little durable action and he urges that physicians do not waste their efforts with these ineffectual technics, but to use the intravenous method, which he claims is much more reliable and durable in its action.

6. Patient could not speak when given treatment. Voice nearly normal in three days. Ulcerations and mucous patches well in 6 days. Could not be cured with mercury and potassium iodid.

He has treated 400 patients; 54 of these received intramuscular and subcutaneous injections with nearly 50 per cent. of recurrences in those treated by these last named methods.

There have been fifteen deaths of adults following the administration of "606," but the fatalities seem to have been due to defects of the patients rather than to the poisonous quality of the remedy. Prof. Rehl, of Vienna, noting the result of 40,000 administrations, thinks that not one death can be ascribed to "606." He further shows that during the last five years 90 fatalities have resulted from injections of mercury.

### PERSONAL EXPERIENCES.

The writer's personal experiences with "606" have been limited to 71 patients, 54 of whom received intravenous injections and the remainder subcutaneous and intramuscular injectons. While the time which has elapsed since the first treatments were administered has been too short to express an opinion as to the ultimate results, the immediate and thorough cure of visible symptoms has been most gratifying both to me and my patients.

No untoward experiences have been observed except a few trivial by-effects and pain at the site of the injections and even this is entirely eliminated in the present technique of administering the remedy by the intravenous route which, to the writer, seems far preferable to the other methods. Only two patients have have had a rise of temperature above 102.5, while the majority have shown subnormal temperature of 1 to 1.5 degrees on the day after the treatment. One patient who received the intravenous injection complained of pain on the inner side of the arm, along the course of the vein and several inches above the site of injection. This developed soon after the treatment and appeared to be of the character of pain and hardness due to a subcutaneous escape of the solution, although this did not seem to be the case—at least, no swelling under the skin could be detected. It appeared more like a periphlebitis and might have been caused by the irritating effect of the solution. The patient, however, made an uneventful recovery and in a week three chancres of two months' duration had healed. The preliminary character of this report prevents a detailed account of the patients treated, but a few of the striking results will be given.

One of the most satisfactory cures was that of a patient with malignant tertiary syphilis. The disease was of three years' duration, during which time nearly every form of medication had been tried, including a course of treatment at Hot Springs. At the time of treatment the patient was in a wretched condition. The left tonsil had been entirely destroyed, and the right one about half destroyed; there was a perforation in the hard palate through into the nose, a gummatous mass blocked the right side of the nose and gave a fullness to this side which was not seen on the left; pain in the throat had been so severe that at times a gargle of cocaine had been used to allay the suffering; there was an ulceration two inches in diameter on the right leg and ulcerous syphilides on the left hand, arm and back.

Jan. 16th, 1911.—.6 gramme of "606" was given subcutaneously, according to the Wechselmann method. The pain was not severe that the patient's throat was so improved the first night that the cocaine gargle was not necessary, nor was its use again required. In three days the mass in the nostrils had melted away like magic and there was a distinct improvement in the patient's feelings. In nine days the ulcer on the leg was healed so that all dressings were discontinued. All the lesions on the skin and mucosa rapidly healed. In three weeks the patient had gained 12 pounds in weight and was well of all symptoms. In five weeks he had gained 21 pounds and was the picture of health.

Another patient with such severe ulcerations in the throat the vocal cords that he could not speak, and with large mucous patches on the tongue, was so improved at the end of 36 hours that he could talk like a patient with a cold and in six days he was well. There was so much laryngeal edema at the time of the treatment that I was afraid to administer the remedy until I had provided an intubation set in his room so that in case a Herxheimer reaction developed, with the momentary intensification of the symptoms, breathing might be accomplished through the tube. There was, however, no occasion to use it.

So uniformly have throat affections responded that I now expect the pain to be relieved or greatly reduced in twenty-four hours, the lesions to become clean and healthy looking in forty-eight and rapid and complete healing at any early date, depending upon the extent of the destruction.

Even with all the wonderful results from a single injection I have never been optimistic as to the chances of curing permanently the disease with a single injection and have recommended that all patients, no matter how splendid the progress take at least another treatment at the end of the month and still others in the future if the Wasserman reaction should remain positive.

It is truly the medical wonder of the age, in spite of its limitations and difficulties in administration.

## Figures and Tables

**Figure f1:**
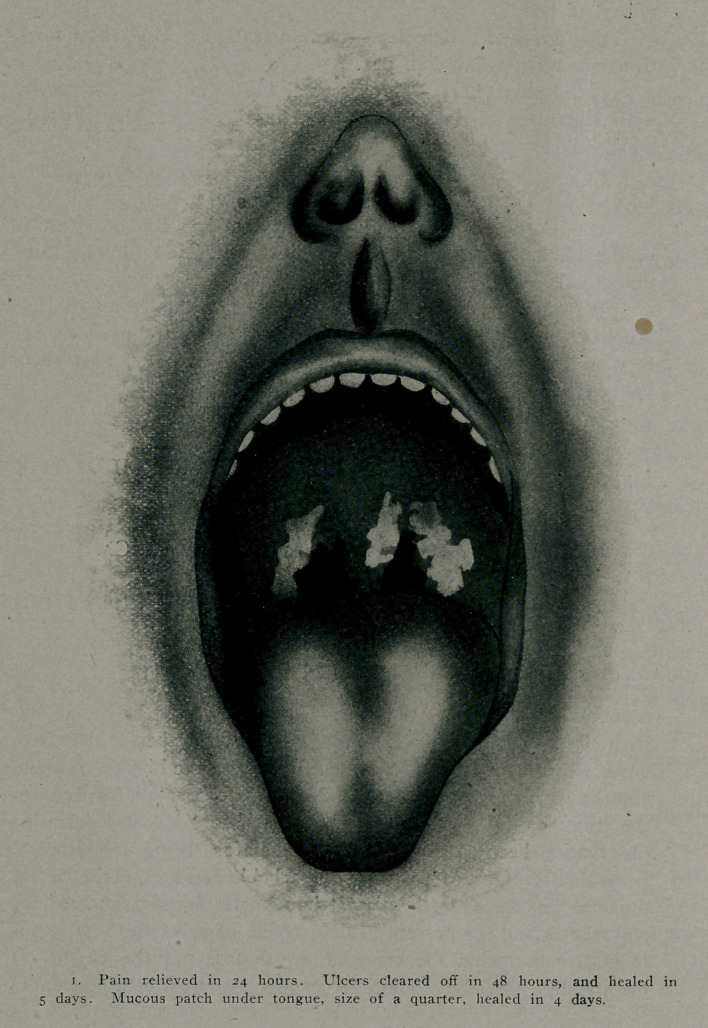


**Figure f2:**
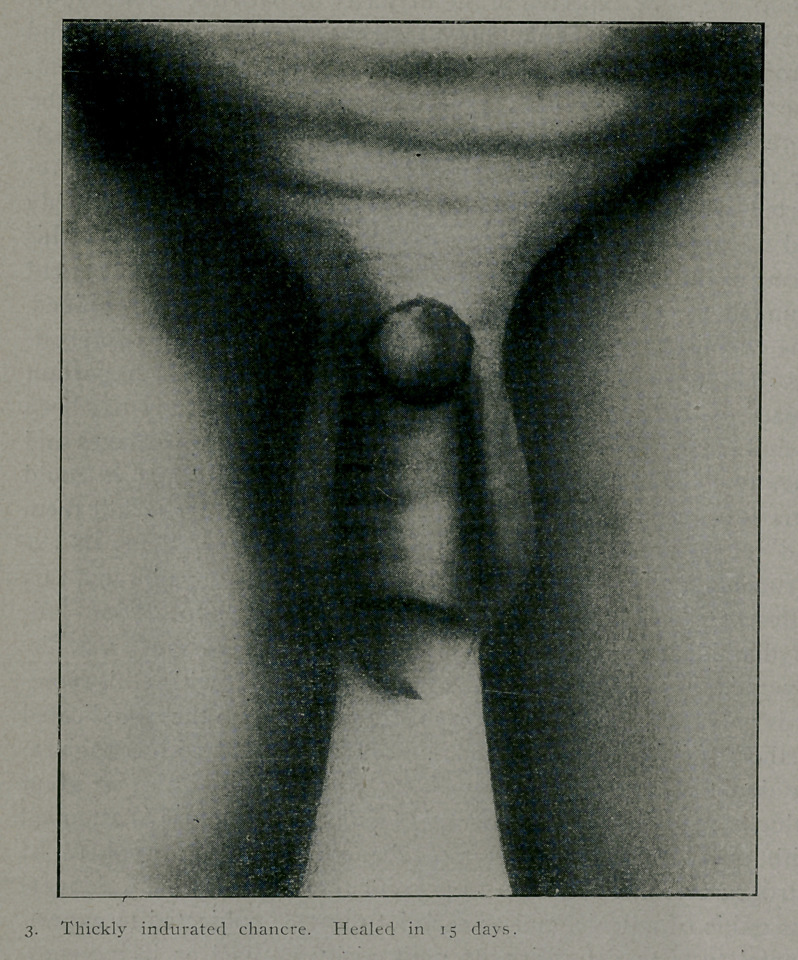


**Figure f3:**
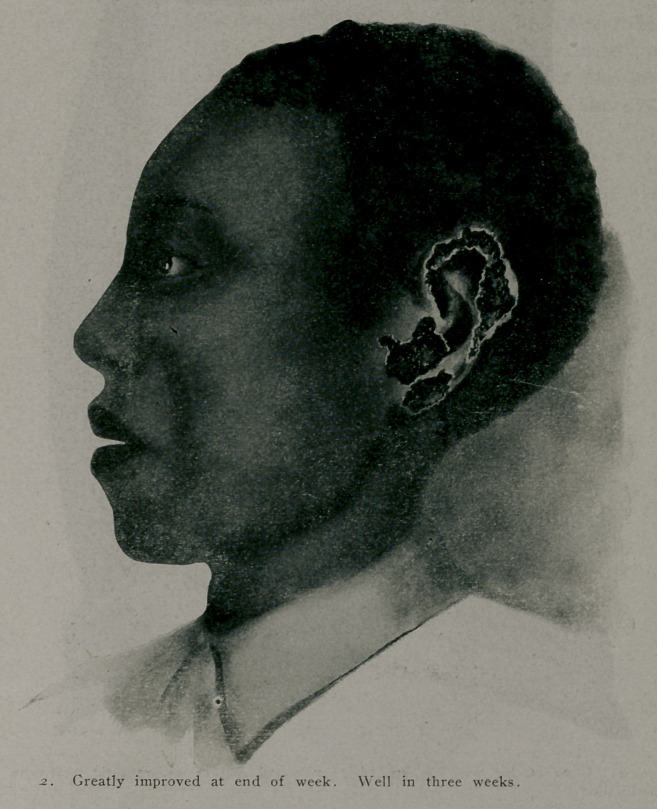


**Figure f4:**
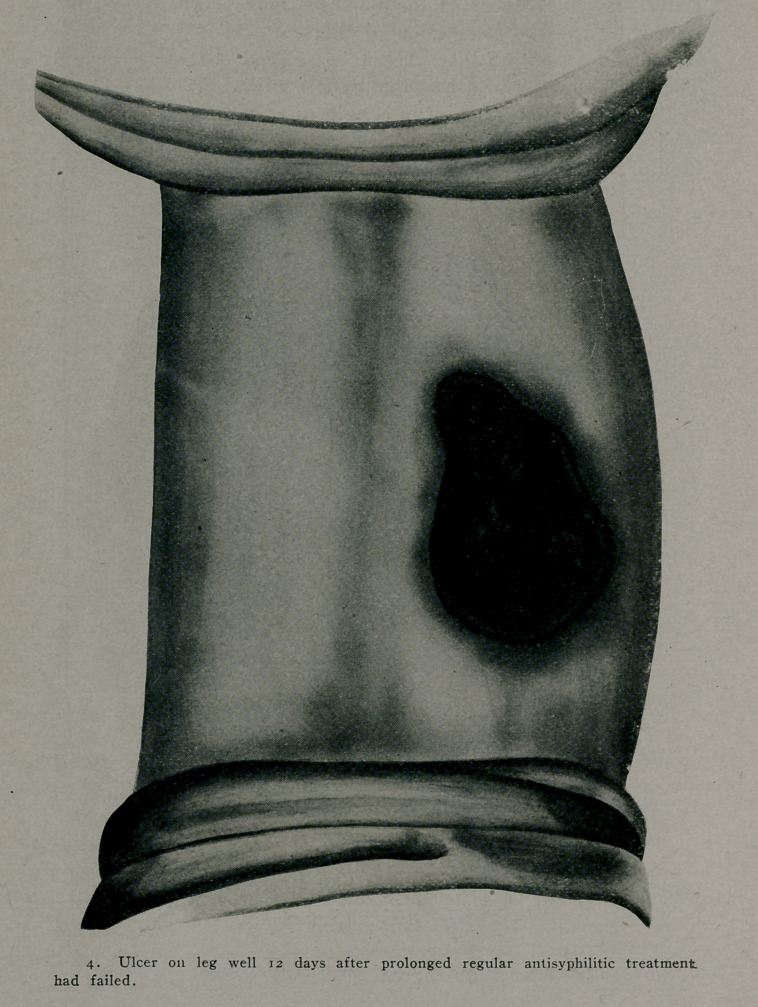


**Figure f5:**
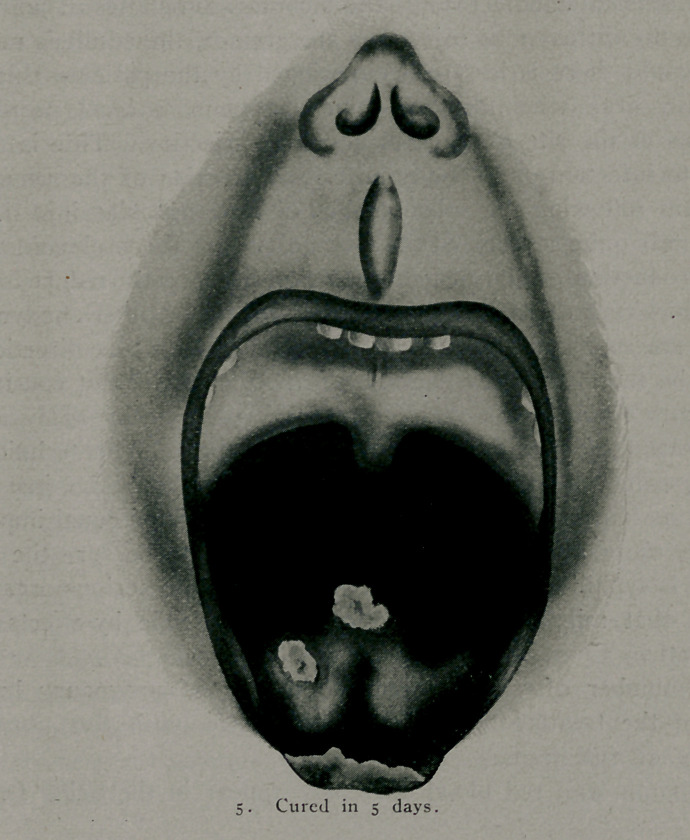


**Figure f6:**